# Fixational Eye Movements in the Earliest Stage of Metazoan Evolution

**DOI:** 10.1371/journal.pone.0066442

**Published:** 2013-06-11

**Authors:** Jan Bielecki, Jens T. Høeg, Anders Garm

**Affiliations:** Marine Biological Section, University of Copenhagen, Copenhagen, Denmark; Lund University, Sweden

## Abstract

All known photoreceptor cells adapt to constant light stimuli, fading the retinal image when exposed to an immobile visual scene. Counter strategies are therefore necessary to prevent blindness, and in mammals this is accomplished by fixational eye movements. Cubomedusae occupy a key position for understanding the evolution of complex visual systems and their eyes are assumedly subject to the same adaptive problems as the vertebrate eye, but lack motor control of their visual system. The morphology of the visual system of cubomedusae ensures a constant orientation of the eyes and a clear division of the visual field, but thereby also a constant retinal image when exposed to stationary visual scenes. Here we show that bell contractions used for swimming in the medusae refresh the retinal image in the upper lens eye of *Tripedalia cystophora*. This strongly suggests that strategies comparable to fixational eye movements have evolved at the earliest metazoan stage to compensate for the intrinsic property of the photoreceptors. Since the timing and amplitude of the rhopalial movements concur with the spatial and temporal resolution of the eye it circumvents the need for post processing in the central nervous system to remove image blur.

## Introduction

All sensory systems desensitise due to receptor adaptation. Visual systems are no different and since photoadaptation occurs at the cellular level of photoreceptors [1]–[4] it is an unavoidable feature in metazoan vision. Thus, all examined photoreceptors adapt to constant visual stimuli [5], and counterstrategies are necessary to prevent image fading or blindness. The best known mechanism to avoid adaptation is the fixational eye movements in mammals (tremor, drift and microsaccades), which continuously refocus and refresh the retinal image [6]–[8]. The movements are generated by an oculomotor system and since they have a blurring effect on the retinal image, additional neural specialisations in post-processing pathways have evolved to eliminate the periods of movement [8]. These mechanisms are very powerful, but also very costly in both energy and neural capacity, and thus, not available for animals with less elaborate processing capabilities.

**Figure 1:**
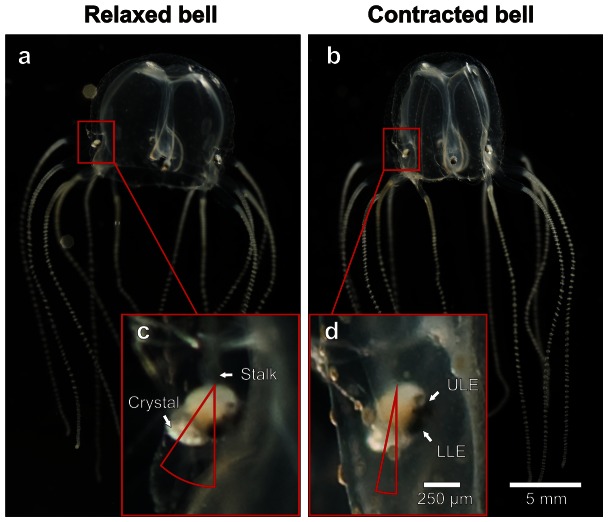
Bell contractions induce rhopalial swing. Due to morphological specializations bell contractions in the box jellyfish *Tripedalia cystophora* make the rhopalia swing between the relaxed state (**a**) and the contracted state (**b**). The corresponding angular change is depicted in **c** and **d**. Video analysis of free swimming animals determined the average swing amplitude of 18–19° to occur within 80–100 ms regardless of the swimming direction (see also Table 1). Here a vertical swimming animal is shown but the results were the same for horizontal and 45° upwards swimming. These values match the known spatio-temporal resolution of the upper lens eye (**d**) suggesting a connection between the biomechanics of the locomotion and refreshing the retinal image. ULE, upper lens eye and LLE, lower lens eye.

Cnidarians were the first of the extant metazoan phyla to develop a nervous system which is therefore considered close to the evolutionary origin of all nervous systems [9]. Within Cnidarians, cubozoans (box jellyfish) have the most elaborate visual system with 24 eyes located on four sensory structures, called rhopalia [10] (Figure 1). In addition to four pigment cup eyes (two pit eyes and two slit eyes), each rhopalium carries two camera-type eyes, morphologically similar to vertebrate eyes, comprising spherical lenses with graded refractive indices. Contrary to the majority of invertebrates, vision in box jellyfish is mediated by ciliated photoreceptors [11], a type normally associated with vertebrate animals. Further, the signal transduction in box jellyfish photoreceptors is based on an opsin-G-protein cascade with a cyclic nucleotide second messenger [12] as in all known ciliated photoreceptors [2]. The strong resemblances to the vertebrate visual system, and their limited nervous system, make box jellyfish ideal models for basic visual information processing.

**Figure 2:**
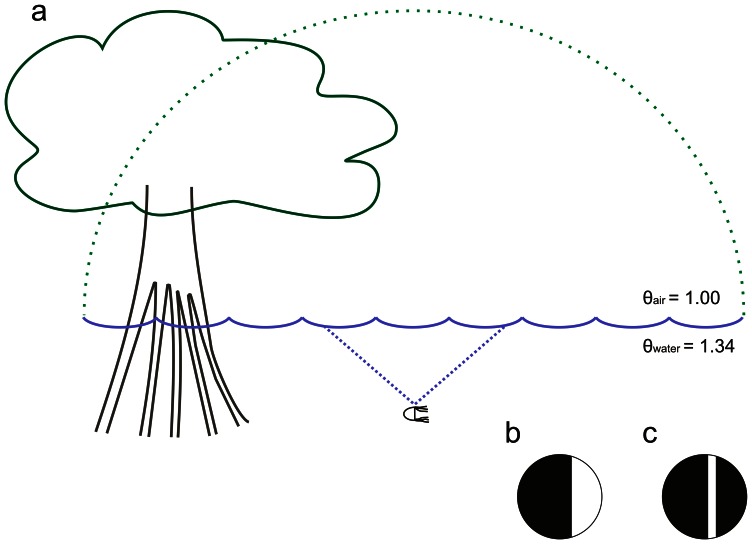
Snell's window. The compression of the 180° horizon above water (green broken line) into a 97° subsurface cone (blue broken line) (**a**) enable the box jelly to long distance navigate back under the mangrove canopy. **b**, the contrast line between the canopy and the open sky as seen through Snell's window (without photoadaptation). **c**, the effect of a swim contraction on the retinal image where activated photoreceptors will light up on a background of inactive receptors.

The box jellyfish are able to extract the necessary information from a complex visual scene that spans a complete sphere around the animal. This is accomplished by special purpose eyes working in concert each with the task of extracting very specific information from the entire visual scene. Unfortunately, little is known about the function of the pigment cup eyes [13], [14]. In contrast, the visual ecology of the lens eyes have been extensively studied and one fundamental aspect, contributing to the lens eye function, is that the rhopalium is suspended from the bell by a stalk and weighted by a heavy calcium sulphate crystal [15] at its distal end. This unique morphology ensures a constant vertical orientation of the rhopalium, regardless of the orientation of the animal [16]. This entails a clear division of the visual field: The upper lens eye (ULE) of *T. cystophora* is directed upwards with a visual field of just less than 100° [16] and the lower lens eye (LLE) is directed downwards into the water with a visual field of 170°. The box jellyfish must navigate a maze of prop roots in the mangrove habitat and since colliding with a root could prove fatal. It is therefore imperative that the animals can stay clear of underwater hazards but at the same time locate light shafts between the roots in which their phototactic prey is located [17]. The visual ecology of the LLE is not yet fully understood but it is evident that this eye controls avoidance and feeding behaviour [18]. Conversely, the function of the upper lens eye is much better described; it gazes up on land through what is known as Snell's window. A phenomenon produced by the difference in refractive indices of air and water causing the visual input from the hemisphere above water to compress into a cone of about 97° under water (Figure 2a). Since the murky water does not offer any directional cues the animals are reliant on the ability of the ULE to detect the mangrove canopy for long distance navigation [16]. The box jellyfish scans Snell's window for the contrast line between the mangrove canopy and the open sky (Figure 2b), and uses the canopy as indication for the direction back to their preferred habitat between the mangrove roots [16]. The navigational task imposes an intriguing problem on the visual system of *T. cystophora*, since adaptation in the photoreceptors should render the medusa blind and unable to detect the canopy. The canopy is a stationary object seen on a relatively long distance (several meters), and if only the speed of the swimming motion is considered, it proves insufficient to refresh the retinal image. The navigational swimming speed of *T. cystophora* is about 5 cm/s which would cause the contrast line to move less than 0.4°/s on the retina of the ULE, at a distance of approximately 5 m from the canopy. Environmental effects are also inadequate to prevent adaptation since the mangrove habitat offers remarkably quiet water where waves are rarely seen and currents only originate from a minimal tide of approximately 30 cm. Furthermore, muscles are not found in association with the eyes [19], which implies that vertebrate type fixational eye movements [6], [8] are not available to the box jellyfish.

We hypothesize the presence of an alternative mechanism to oculomotor capabilities where the bell contractions during swimming will cause the entire eye bearing rhopalium to swing and thereby refresh the retinal image. The bell contractions are likely to cause the rhopalium to swing in a pendular fashion and if this swinging is of a magnitude comparable to the receptive angles of the photoreceptors, the locomotion of the animal could be used as an indirect mechanism to prevent adaptation in the upper lens eye.

## Results

**Figure 3:**
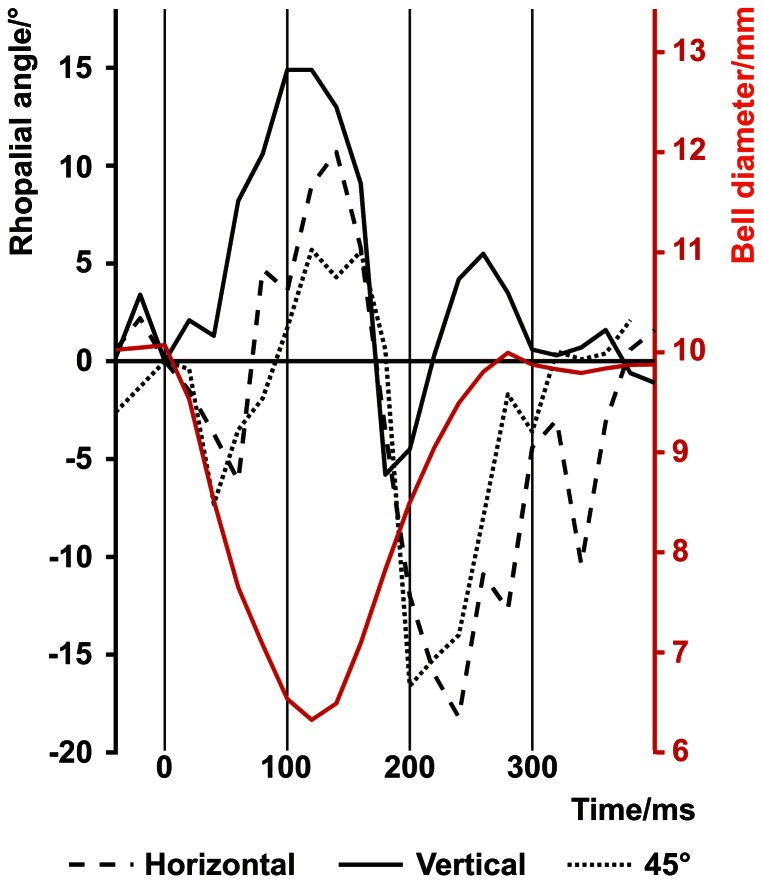
Variation in rhopalial swing pattern. Tracking the rhopalial angle through a single bell contraction show variations in the swing pattern, but the swing amplitude and swing time remain relatively constant (see Table 1). The tracings are synchronized in relation to the onset of the bell contraction, and a contraction of each of the swimming directions is shown. Red trace is the average bell diameter for the three contractions.

**Figure 4:**
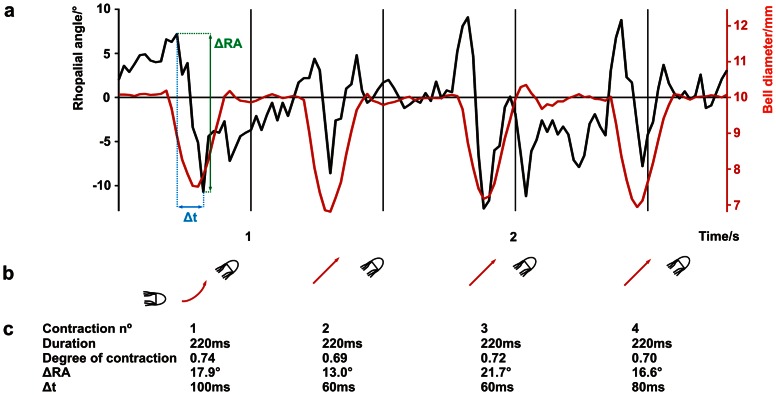
Tracking the rhopalial swing amplitude. Tracking the amplitude of the rhopalial swing, ΔRA, and the swing time, Δt, in a series of swim contractions (**a**) showed deviation from the hypothesized sinusoidal swing pattern but most contractions induced a rhopalial swing which matches the spatio-temporal resolution of the upper lens eye (**c**). It is evident that the bell contraction (**a**, red trace) induced the rhopalial swing (**a**, black trace). The pictograms in **b** represent the orientation of the animal and, red arrows, the swimming direction for each of the tracked contractions. The contraction parameters (duration, degree of contraction, ΔRA and Δt) remained relatively constant (**c**).

To test our hypothesis we did macro video recordings of freely swimming animals and they revealed that the swim contractions did indeed cause the rhopalia to swing. The detailed analysis of the swim mechanics showed the average angular shift of the rhopalium (ΔRA) induced by the bell contraction (Figure 1) to be 18.5°±0.5°. The full amplitude of the swing was completed within a timeframe (Δt) of 86 ms±5 ms. Interestingly, ΔRA and Δt remained constant irrespective of the swimming direction of the animal and variations in the swing pattern (Figures 3 and 4). The values of ΔRA were 19°, 18.1° and 18.5° for horizontal, vertical and 45° upward swimming respectively and Δt values 80 ms, 89 ms and 89 ms for horizontal, vertical and 45° upward swimming respectively (Table 1).

**Table 1:** Rhopalial displacement induced by bell contractions.

Swimming direction	Mean ΔRA ±SEM	Mean Δt ±SEM
Horizontal	19±1.2°	80±7 ms
Vertical	18.1±0.7°	89±5 ms
45° upward	18.5±0.6°	89±7 ms

The swing amplitude (ΔRA) and time (Δt) of the rhopalium varies little with the direction of swimming. ΔRA and Δt match the known spatio-temporal resolution of the upper lens eye. Mean ± standard error of the mean, N = 30.

**Figure 5:**
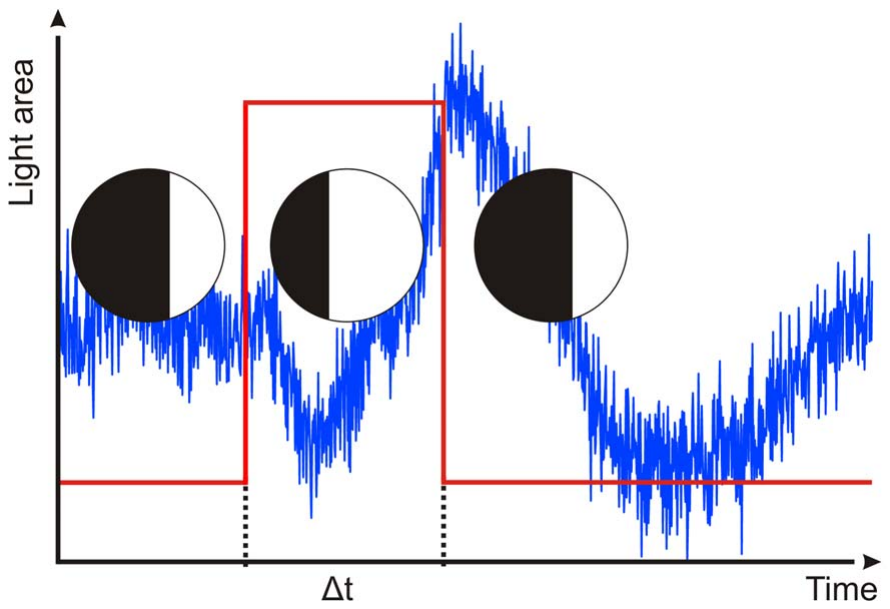
Imitating the swim contractions. Light area in the circles represents the amount of open sky in the visual field. The upper lens eye was exposed to a changing area mimicking the effect of a swim contraction (red trace). Blue trace depicts a typical ERG response to the change in light area of the fiber optic image bundle.

**Figure 6:**
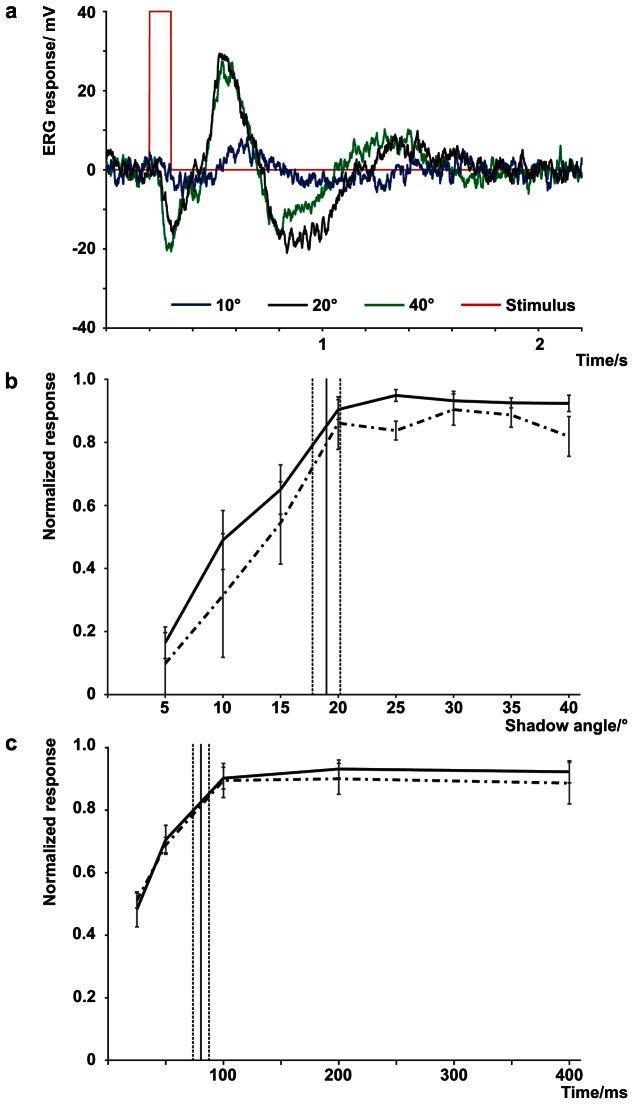
Electroretinogram response to a moving contrast line. The electroretinogram (ERG) confirmed the finding that the bell contractions refreshed the retinal image. Even though all changes in light are registered by the photoreceptors, the optimal response and, thereby optimal image contrast, was obtained when the change matched the photoreceptor acceptance angle (**a**). A shadow was moved in the visual field of the upper lens eye to mimic the contrast line between the mangrove canopy and the open sky, and manipulated to test the neural response to different rhopalial swing amplitudes. There was a marked increase in the ERG response up to a 20° displacement of the shadow after which no significant change in ERG amplitude was detected (**b**). A similar result was obtained when manipulating the swing time (**c**); here the ERG amplitude did not increase further when the shadow displacement time was longer than 100 ms. These data match the known spatio-temporal resolution of the ULE and strongly support the hypothesis that the bell contractions refresh the retinal image in the upper lens eye of *T. Cystophora*. Vertical black lines in **b** and **c** represent the amplitude and time of the rhopalial swing measured in a horizontal swimming animal, which is preferred swimming direction of the animal for navigational purposes (interrupted lines, ± standard deviation of the mean). The experiments were performed at two light intensities (solid line, 30 Wm^−2^sr^−1^ and interrupted line, 10 Wm^−2^sr^−1^, ± standard deviation of the mean, N = 8).

To substantiate the behavioural data we conducted a series of electrophysiological experiments to confirm the functional significance of the rhopalial swing. Here the upper lens eye was exposed to a moving shadow in the visual field designed to mimic the contrast line between the mangrove canopy and the open sky (Figure 5). Only dark-adapted photoreceptors that were exposed to light in the experimental protocol produced a response, whereas light adapted photoreceptors did not respond to a change in light intensity. The shadow was manipulated to simulate a rhopalial swing with amplitudes between 5° and 40° in steps of 5° which were all completed in 100 ms. We also tested the temporal component of the swing by changing the duration of the moving shadow (25, 50, 100, 200, and 400 ms), all with the same amplitude of 20°. We monitored the physiological response from the photoreceptors and possibly also some higher order neurons by electroretinogram (ERG) recordings (Figure 6a). Moving the shadow within the visual field of the upper lens eye produced a graded response typical of extracellular recordings from photoreceptors [20] (Figure 6a). The ERG response increased 5-fold when increasing the swing amplitude from 5° to 20°, but increasing the swing further had no additional effect (Figure 6b). Similarly, the ERG response increased almost two-fold when manipulating the temporal component from 25 to 100 ms, where after the response again reached a plateau (Figure 6c). We performed the entire protocol at two different intensities (10 Wm^−2^sr^−1^ and 30 Wm^−2^sr^−1^), and while the ERG responses were generally slightly smaller in the lower intensity, approximately 5 %, the relative changes were the same (Figure 6 b and c).

## Discussion

Morphological specialisations in the visual system of the box jellyfish ensure a constant vertical orientation of the visual field of the eyes [10], [16], [21], but the stability of the system makes the animals vulnerable to image fading of stationary objects [6], [7]. It has never been unequivocally proven that photoadaptation occur in cubozoan photoreceptors, but since it is a general feature of all known ciliated photoreceptors [1], [5] and box jellyfish photoreceptors work through an opsin- Gs-protein cascade [12], we assume it to be the case. *Tripedalia cystophora* relies on terrestrial visual cues to navigate back to their preferred habitat under the mangrove canopy [16] by detecting the contrast line between the canopy and the open sky (Figure 2b). The contrast line is stationary and the inability to detect it could prove fatal for the animal. Through behavioural and extracellular electrophysiological experiments we have found that box jellyfish utilize the swim contractions to counteract the putative image fading in the upper lens eye. Traditionally eye evolution has been viewed as an optimization of the inverse relationship between spatial resolution and light sensitivity [22]. But a parallel to fixational eye movements in this basal metazoan group could indicate that photoadaptation has exerted additional selective pressure on the evolution of visual systems.

For the swim contractions to function efficiently as fixational eye movements they must induce a change to the retinal image of a magnitude that shifts the visual scene one half width of the photoreceptor acceptance angle within a single integration time. We observed large variations in the pattern of the rhopalial swing (Figure 3) in response to a rather invariable bell contraction, yet they resulted in relatively constant swing amplitudes and durations regardless of the orientation of the animal itself (Table 1). These consistencies suggest a functional significance and since swing amplitudes are much larger during bell contractions than in the inactive periods between bell contractions where the animal glides through the water (Figure 4) only the contractions could potentially refresh the retinal image.

The optimal ERG response from the photoreceptors occurred when displacing the shadow 20° within 100 ms which matches our behavioural results (Figure 6) and the findings of Nilsson et al (2005) [11] that the approximately 400 photoreceptors which make up the retina of the upper lens eye in *T. cystophora* have acceptance angles with half widths varying from 10 to 20° depending on their location in the retina. A curious aspect of *T. cystophora* vision contributes to a rather poor image quality; the lens of the camera-type eyes do not focus the image on the retina but rather a distance behind it [11]. But it is only due to this blurry image, with a spatial resolution of 10–20°, that a crude system such as swim contractions can function as fixational eye movements. Had the box jellyfish retinal image been of the same acuity as in human eyes, the inaccuracy of the rhopalial swing amplitude would have contributed to image blur rather than preventing it. There is also a close match with the known integration time, which varies with light intensity, but remains within the magnitude of 100 ms for the intensities used here [23]. The two intensities used in this study (10 Wm^−2^sr^−1^ and 30 Wm^−2^sr^−1^) produced the same relative ERG response to the imposed ΔRA and Δt, suggesting a very robust system that works across a range of light intensities.

Physiologically it is important that there is a close correlation between the spatio-temporal resolution of the photoreceptors and the fixational eye movements of the box jellyfish. Photoadaptation will be counteracted by any movement of the eye, but if the movement is too small or too fast sub-optimal contrast will be achieved. If the movement is too large or too slow unnecessary blur will be added to the image without additional gain in counter adaptation and thereby contrast. Taken together, the constancy in amplitude and timing of the swing and the strong correlation with the known spatio-temporal resolution of the upper lens eye allow us to speculate that bell contractions, in addition to serving for locomotion, in the box jellyfish *T. cystophora*, function as a parallel to the fixational eye movements in mammals, which refresh the retinal image with minimal loss in visual acuity. In an adapted photoreceptor the change in photon capture, caused by movement, will automatically initiate an integration time, the duration of which is set by the contrast (relative change in photon capture) achieved by the movement. The amplitude of the photoreceptor response is directly correlated with the perceived contrast of the visual image [24], and the retinal photoreceptors of the ULE, which experience the greatest intensity change within a swing, will naturally be the most affected. The contrast line between the canopy and the sky will light up (activated photoreceptors) on a dark background (inactive photoreceptors) (Figure 2c). Thereby the animal has filtered the information needed for navigation from a more complex visual scene as early as the retinal level and circumvented the need for vertebrate-like neural processing in the CNS to remove image blur, which likely would be beyond the neural capacity of cubomedusae.

The eye bearing rhopalium is a rigid structure, which implies that all the eye types experience the same swing amplitude. This work focused on the ULE since the visual ecology of the other eyes is less established. It is known that the lower lens eye is involved in obstacle avoidance behavior [18] but it is not clear whether swim contractions are necessary for detecting the prop roots under water or if the swimming speed itself is sufficient for detecting nearby structures.

Not only mammals and box jellyfish have evolved mechanisms the refresh the retinal image. The ctenid spider, *Cupiennius salei*, has spontaneous retinal microsaccades of approx. 12 Hz to prevent photoadaptation in the antero-median eyes. This system compares to the swim contractions of *T. cystophora* in that the saccades correspond to the spatio-temporal resolution of the spider eyes [25]. Interestingly, the spider might use photoadaptation to their advantage in their postero-lateral eyes. By not counteracting the photoadaptation these eyes are thought to be used in movement detection only [26], allowing all stationary objects to fade from the perceived image. We suggest an even more ingenious and efficient system to be present in the box jellyfish, where stationary objects will gain contrast during pulsing, as shown here (Figure 2c), while pauses in swimming [27] will enhance moving objects on a stationary background. Both stationary and moving objects will be detected in the same eye but filtered by the innate behavior of the animal respectively. For animals with limited neural processing capacity, such simple matched filters [28] are probably of great importance in order for them to perform their seemingly advanced visually-guided behaviours.

In conclusion, our results suggest that a fixational eye movement strategy is necessary to detect stationary objects not only in mammals but throughout Metazoa. Box jellyfish can utilize an already operational system by having the swim contractions refresh the retinal image in the upper lens eye. These findings point to an often overlooked aspect in the evolution of visual systems. Eye evolution has largely been considered driven by optimization of the inverse relationship between light sensitivity and spatial resolution in order for the animals to maximize their visual performance [22]. However, if fixational eye movements counteracting photoadaptation are present already in the most basal animal group with image-forming eyes, the box jellyfish, our findings suggest that photoadaptation also has exerted severe selection pressures that should be considered in the evolution of visual systems. If eyes are not accompanied by a system to counteract photoadaptation they will be less suitable in detecting stationary objects, even with the most advanced optics.

## Materials and Methods

### Animals

The experiments were performed on adult medusae (approx. 10 mm in bell diameter) of *Tripedalia cystophora* Conant 1897 obtained from our cultures at the University of Copenhagen, Denmark. In the cultures the medusae were kept in a 200 l tank with circulating seawater at 28‰ and about 28°C and fed SELCO (INVE Technologies, Dendermonde, Belgium)-enriched Artemia daily. They reached adult size in 2–3 months.

### Video Analysis

To minimise the animal's movements in the plane parallel to the direction of filming the medusae were transferred from the culture tank to a transparent aquarium (inside dimensions w∶d∶h; 20∶2∶11 cm). Bell contractions were filmed using a video camera (DXC-950P, Sony Corp, Tokyo, Japan) fitted with a Nikon 105 mm macro objective (shutter speed 1/500 s, f8–f11). Sufficient lighting for adequate picture quality were supplied by four 100 W Philips soft tone light bulbs. Single frames were grabbed using Pinnacle Studio software, transferred into Corel Draw (version X13 Corel Corp, Ottawa, Canada) and deinterlaced into separate fields, thereby producing a time resolution of 20 ms. The angle at which the rhopalium was suspended from the bell was determined by a line through two rhopalial fix points (midpoint of stalk attachment and midpoint of the crystalline weight) compared to vertical (Figure 1). The rhopalial angle, as well as the bell diameter was determined in each field. The rhopalial movement and the corresponding contraction time were determined for three swimming directions: horizontal, vertical and approximately 45° upwards (Figure 3). Three contractions of each direction were analyzed from ten animals. Only contractions where the rhopalial movement was directly perpendicular to the direction of filming were analyzed to avoid discrepancies due to movement in the third plane. The rhopalial angle and the bell diameter were plotted against time and the maximum amplitude of the rhopalial swinging (ΔRA) within the timeframe (Δt) was recorded for each contraction (Figure 3 and 4). To test whether the rhopalia were swinging between contractions due to other influences, the rhopalial angle were analysed through a series of contractions (Figure 4).

### Electroretinogram (ERG)

The recording procedures and animal handling strictly followed the experimental setup described in O′Connor et al (2010)[23]. The rhopalium was oriented with the ULE directed upward and a fiber optic image bundle (extracted from an Olympus CF-100TL endoscope) was placed such that the image covered 90° of the visual field of the ULE. At the distal end of the light guide a Luxeon V star LED (LXHL-LW6C, cool white, Lumileds Lighting, LLC, San Jose, CA, USA) mounted in an optical bench (Linus, Leiden, Germany) ensured an even illumination of the entire light guide surface. A piece of black cardboard was mounted on an outlet speaker membrane and placed between the light source and the distal end of the light guide so that it covered approximately ¾ of the surface area. The experimental setup was manipulated until the shadow displacement affected the area on the retina immediately above the suction electrode. The amplitude (square waves) and speed of the speaker membrane was controlled by LabVIEW 8.5 software and the same DAQCard-NI6229 data acquisition card used for recording (both National Instruments, Austin, TX, USA). The shadow was manipulated to simulate various swing amplitudes by exposing the ULE to shadow displacement in 5° increments from 5 to 40° within 25–400 ms. Angular tests ran at a shadow displacement time of 100 ms and swing time tests with a displacement of 20° and the entire experimental protocol was performed at two light intensities (30 Wm^−2^sr^−1^ and 10 Wm^−2^sr^−1^). Eight rhopalia from adult animals were used for the experiments, and each rhopalium was exposed to the entire protocol (all angles, shadow times and intensities). The experiments were concluded within one hour after sectioning.

## Supporting Information

Movie S1 **Video recording of the rhopalial swing.** The rhopalial angle (yellow interrupted line) were tracked through a bell contraction and compared to the angle at the relaxed, or t_0_, position (red interrupted line). The video was slowed to 2 frames per second.
(WMV)
